# Balkan endemic nephropathy: an update on its aetiology

**DOI:** 10.1007/s00204-016-1819-3

**Published:** 2016-08-19

**Authors:** Marie Stiborová, Volker M. Arlt, Heinz H. Schmeiser

**Affiliations:** 1 Department of Biochemistry, Faculty of Science, Charles University, Albertov 2030, 128 40 Prague 2, Czech Republic; 2 Analytical and Environmental Sciences Division, MRC-PHE Centre for Environmental and Health, King’s College London, 150 Stamford Street, London, SE1 9NH UK; 3 NIHR Health Protection Research Unit in Health Impact of Environmental Hazards at King’s College London in partnership with Public Health England, Franklin–Wilkins Building, 150 Stamford Street, London, SE1 9NH UK; 4 Division of Radiopharmaceutical Chemistry (E030), German Cancer Research Center (DKFZ), Im Neuenheimer Feld 280, 69120 Heidelberg, Germany

**Keywords:** Balkan endemic nephropathy, Disease aetiology, Upper urothelial cancer, Environmental and genetic factors, Aristolochic acid nephropathy, Aristolochic acid

## Abstract

Balkan endemic nephropathy (BEN) is a unique, chronic renal disease frequently associated with upper urothelial cancer (UUC). It only affects residents of specific farming villages located along tributaries of the Danube River in Bosnia-Herzegovina, Croatia, Macedonia, Serbia, Bulgaria, and Romania where it is estimated that ~100,000 individuals are at risk of BEN, while ~25,000 have the disease. This review summarises current findings on the aetiology of BEN. Over the last 50 years, several hypotheses on the cause of BEN have been formulated, including mycotoxins, heavy metals, viruses, and trace-element insufficiencies. However, recent molecular epidemiological studies provide a strong case that chronic dietary exposure to aristolochic acid (AA) a principal component of *Aristolochia clematitis* which grows as a weed in the wheat fields of the endemic regions is the cause of BEN and associated UUC. One of the still enigmatic features of BEN that need to be resolved is why the prevalence of BEN is only 3–7 %. This suggests that individual genetic susceptibilities to AA exist in humans. In fact dietary ingestion of AA along with individual genetic susceptibility provides a scenario that plausibly can explain all the peculiarities of BEN such as geographical distribution and high risk of urothelial cancer. For the countries harbouring BEN implementing public health measures to avoid AA exposure is of the utmost importance because this seems to be the best way to eradicate this once mysterious disease to which the residents of BEN villages have been completely and utterly at mercy for so long.

## Introduction

**Fig. 1:**
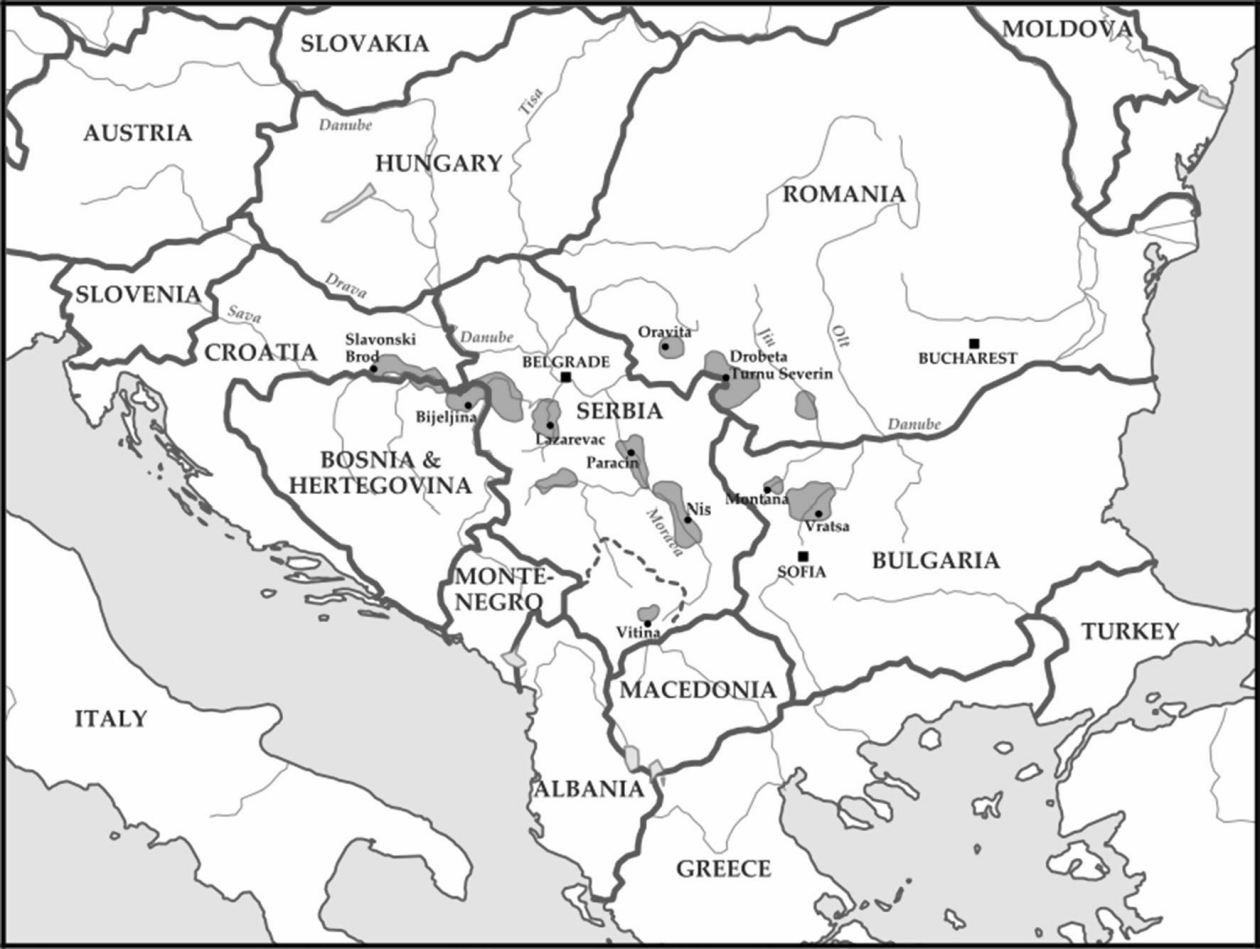
Distribution of BEN foci in Bosnia-Herzegovina, Croatia, Serbia, Bulgaria, and Romania (https://en.wikipedia.org/wiki/Danubian_endemic_familial_nephropathy#/media/File:Balkan_endemic_nephropathy_map.svg)

Balkan endemic nephropathy (BEN) is a chronic tubulointerstitial nephropathy characterised by an insidious onset and gradual progression to end-stage renal disease (ESRD) which was first described more than 60 years ago (Danilovic et al. 1957; Tanchev et al. 1956). The disease affects residents of rural farming villages located along the tributaries of the Danube River in Bosnia-Herzegovina, Croatia, Macedonia, Serbia, Bulgaria, and Romania (Fig. 1) (Grollman 2013; Pavlovic 2013; Pfohl-Leszkowicz 2009; Radovanović 2002; Stefanovic 1983).

A characteristic feature of BEN is its close association with upper urothelial cancer (UUC) of the renal pelvis and ureter (Miletić-Medved et al. 2005; Radovanovic 2002; Stefanovic 1983; Stefanovic and Radovanovic 2008). These UUCs are mostly carcinomas (Toncheva et al. 2014) and are the most common causes of death in BEN patients. BEN patients also have a higher risk of developing UUC after kidney transplantation (Basic-Jukic et al. 2007). The difference in the prevalence of UUC between the general kidney transplant population (0.69 %) and the BEN population (43.7 %) is enormous, confirming the association of BEN with UUC. In BEN patients, bilateral nephroureterectomy before transplantation has been suggested as a preventive measure (reviewed in Stefanovic et al. 2015).

## Epidemiology, pathology, clinical features and diagnosis of BEN

The most remarkable feature of BEN is its endemic nature and its familial but not inherited pattern of distribution. Nevertheless, besides the endemic regions shown in Fig. 1, sporadic cases of BEN were also found in other regions (Stefanovic et al. 2009, 2015). BEN only affects the rural farming population, but has never been found among inhabitants of larger cities. The disease exhibits a focal occurrence within certain villages where remarkably affected villages are often in close proximity to unaffected villages (Bamias and Boletis 2008). Moreover, within the same village, affected and spared households often live in close proximity. In a single household, numerous members of one or several generations can be affected (Stefanovic et al. 2015); therefore, it has been postulated that only persons “living under the same roof and eating the same food” are at risk (Bamias and Boletis, 2008). This peculiar geographic distribution has remained constant since the 1950s.

BEN has an onset of disease between 40 and 60 years of age and has a long latency period. Individuals of both genders are affected, with slight female predominance. The disease occurs in adults, but not in children (Grollman 2013). BEN frequently results in terminal kidney failure, and it has been estimated that ~100,000 individuals are at risk, while ~25,000 have the disease (Pavlovic 2013).

The pathology of BEN has been reviewed in numerous reports (Bamias and Boletis 2008; Jankovic et al. 2009; Jelakovic et al. 2014; Pavlovic 2013; Schiller et al. 2008; Wernerson et al. 2014). The pathology of BEN shares similarities with tubulointerstitial renal diseases and is characterised by progressive kidney atrophy and sclerosis (Pavlovic 2013). Histologically, BEN is characterised by extensive hypocellular interstitial fibrosis associated with tubular atrophy. Glomerular and vascular lesions are associated with periglomerular fibrosis, glomerular lesions, including ischaemic, microcystic, obsolescent glomeruli, occasional thrombotic microangiopathy-like lesions and focal segmental sclerosis-like lesions (Pavlovic 2013).

The clinical symptoms and markers of BEN are not specific and frequently remain unrecognised for several years (Radonić and Radosević 1992; Stefanovic et al. 2009). After an initial asymptomatic stage, patients suffer from weakness and lassitude, mild lumbar pain, pallor of the skin and a copper-brownish discoloration of the palms and soles. At this phase of the disease, occurring usually at an older age (Cukuranovic et al. 2007; Djukanović et al. 2007), anaemia develops which is associated with a significant loss of renal function indicating the presence of chronic kidney disease (CKD). Intermittent proteinuria indicating proximal tubular damage can be found early, whereas in the uraemic stage of BEN, it becomes permanent. Loss of urine concentration capacity precedes a decrease in the glomerular filtration rate (Alecković et al. 2010; Arsenović et al. 2005; Dimitrov et al. 2006).

Kidney atrophy has been suggested as one of the criteria for the clinical diagnosis of BEN. However, investigations of kidney status have shown a variable decrease in kidney size with very small contracted kidneys in ESRD (Radonić and Radosević 1992). Unfortunately, there are no diagnostic markers of BEN, which are characteristic for the disease. However, the set of epidemiological, clinical and biochemical data along with the pattern of pathologic injury, in the absence of any other renal disease, is highly suggestive of BEN in affected countries. The diagnostic criteria for BEN were described for the first time more than 50 years ago and have been improved continuously since then. However, criteria differed in affected countries and a meeting was held in Zagreb, Croatia, in 2006 (Grollman and Jelakovic 2007) to establish more unified diagnostic criteria for BEN. Thereafter, an international panel of researchers has agreed on appropriate criteria for epidemiologic and clinical studies on BEN (Stefanovic et al. 2007).

During the “International Workshop on the Diagnostic Criteria in BEN”, held in Brač, Croatia, in 2008, and at a meeting organised in 2012 in Skopje, Macedonia, novel criteria were evaluated and used for preparation of up-dated recommendations (KDIGO 2013). The consensus was targeted to provide recommendations for the screening, diagnosis and therapy of patients suffering from BEN (Jelakovic et al. 2014). A recent study by Dika et al. (2014) evaluated the diagnostic significance of the variables previously proposed to unify the diagnostic criteria of BEN, but also included new criteria. In the study patient subgroups, no statistical differences in haemoglobin level, leucine aminopeptidase in urine and active urinary levels of transforming growth factor β were found in the BEN diseased group when compared to other subgroups. Kidney length and parenchyma thickness, α1-microglobulinuria and kidney function assessed by the Modification of Diet in Renal Disease (MDRD) formula were the variables that differentiated the study subgroups of patients. Based on these results, the cut-off value of α1-microglobulin for screening was considered to be 23.5 mg/g creatinine and for making a diagnosis of BEN 31.5 mg/g creatinine. However, thus far no serum or urinary biomarkers have been shown to be really useful to clinically diagnose BEN.

## Hypotheses of BEN aetiology

Soon after the first description of BEN, investigations on its cause were started. Probably no other human disease has produced so many hypotheses to resolve its aetiology.

To date several hypotheses have been formulated that could be relevant to the aetiology of BEN. These hypotheses can be divided into two groups; the first group represents exogenous environmental factors and the second group includes confounding factors. It should be noted, however, that only one of the hypotheses, namely the chronic poisoning with aristolochic acid (AA), a toxin produced by plants of the genus *Aristolochia*, has provided convincing evidence to be the primary causative agent in BEN, and particular in the role of developing BEN-associated cancer. Indeed, environmental exposure to AA by BEN patients is now well documented (Anandagoda and Lord 2015; Arlt et al. 2002a, 2007; Bamias and Boletis 2008; Bui-Klimke and Wu 2014; Grollman 2013; Grollman et al. 2007; Jelakovic et al. 2014; Pavlovic 2013; Schmeiser et al. 2012; Stefanovic et al. 2015)]. Therefore, the review focuses on the role of AA in the aetiology of BEN and associated cancer.

## Exogenous factors relevant for BEN aetiology

For the past decades a variety of environmental agents have been investigated (Arlt et al. 2002a, b, 2007; Bamias and Boletis 2008; Batuman 2006; Grollman 2013; Ivić 1969; Pfohl-Leszkowicz et al. 2002, 2007, 2009; Radovanovic 2002; Stefanovic and Cosyns 2005; Stefanovic et al. 2015; Voice et al. 2006), and among them various heavy metals or metaloids, mycotoxins [in particular ochratoxin A (OTA)], organic chemicals from Pliocene lignite deposits located in endemic areas in the Balkans (hydrogeochemical factors) and the nephrotoxic and carcinogenic plant product AA.

### Metals and metalloids

Concerning several metals or metalloids, it has been hypothesised that either their low or high concentrations can mediate the development of BEN. The hypothesis was based on the finding that some of them, such as silica, lead, uranium, copper, cobalt, zinc, manganese, arsenic, titanium, barium, aluminium, chromium, strontium, cadmium, bismuth, molybdenum, nickel, tungsten, antimony, and tin can be present in water and soil in BEN areas (Bui-Klimke and Wu 2014). BEN has been suggested to be associated with high levels of these elements in water (Nichifor et al. 1985) and tumour formation has been linked to exposure to silica and nickel (Markovic et al. 1976). In contrast, metal analysis in water and soil in BEN areas have found that measured concentrations all fell below the detection limits of the analytical methods used (Pfohl-Leszkowicz et al. 2002). Further, results found in a study by Karmaus et al. (2008) indicated that metals such as cadmium, lead, metalloids arsenic and selenium do not play a critical role in the aetiology of BEN.

The concentrations and the extent of selenium deficiency are well documented in rocks, soil, water, food stuffs, and serum samples collected from endemic and non-endemic regions of BEN in Serbia (Maksimovic 1991; Maksimovic et al. 1992; Maksimovic and Djujic 1997). However, there was inadequate evidence that selenium deficiency was a causative agent for BEN and UUC in endemic areas (Maksimovic 1991; Maksimovic et al. 1992; Maksimovic and Djujic 1997). Indeed, a later review by Batuman (2006) reported that selenium was uniformly distributed between endemic and non-endemic areas and was highly improbable to be a cause of BEN.

All these findings led to conclusion to rule out heavy metals and metalloids from the causes of BEN (Batuman 2006).

### Ochratoxin A

The ochratoxin A (OTA) hypothesis was based on the fact that residents in endemic regions are exposed to relatively high concentrations of OTA (Radić et al. 1997). It was one of the first well-elaborated hypotheses regarding the aetiology and pathogenesis of BEN, which was described in the 1970s (Krogh et al. 1977). Several studies carried out in various areas of the world including many countries in Europe have shown that the mycotoxin OTA is a natural contaminant of many plant products. OTA-mediated nephropathy is endemic, and outbreaks have been associated with weather conditions (Hald 1991). Nevertheless, similar high exposure to OTA occurs throughout the world in farming communities that are largely free of CKD and urothelial malignancy (De Broe 2012).

OTA is a potent nephrotoxin and renal carcinogen in rodents. Although this mycotoxin is considered to be a possibly carcinogenic to humans (Group 2B as classified by International Agency for Research on Cancer [IARC]), it has never been linked to any nephropathy in humans (Stefanovic et al. 2015). An endemic nephropathy, which showed clinical and pathological similarities with BEN, was observed in Tunisia, where some regions are contaminated with OTA (Maaroufi et al. 1995). Nevertheless, chronic nephrotoxicity clearly associated with dietary exposure to OTA has not been observed in humans (Godin et al. 1997). Furthermore, the mechanism of OTA-derived tumour formation is unknown, and conflicting results regarding the potential of OTA to react with DNA to form covalent DNA adducts have been reported (Castegnaro et al. 2006; Mally and Dekant 2005, 2009; Manderville 2005; Mantle et al. 2010; Pfohl-Leszkowicz and Manderville 2012; Turesky 2005). Based on positive results detecting DNA adduct spots by the ^32^P-postlabelling method, some investigators postulated that OTA forms covalent DNA adducts (Pfohl-Leszkowicz et al. 1991, 1993), whereas several in vitro and in vivo studies using radiolabeled OTA consistently failed to detect radioactivity associated with DNA (Gautier et al. 2001; Gross-Steinmeyer et al. 2002; Mally et al. 2004; Schlatter et al. 1996). These findings suggest that the adduct spots detected by ^32^P-postlabelling may not contain OTA or parts of the OTA molecule. Taking into consideration all the available data, the European Food Safety Authority (EFSA) scientific panel on contaminants in the food chain concluded that there was no evidence for the existence of specific OTA-DNA adducts and that the genotoxic effects of OTA were most likely attributable to oxidative stress (European Food Safety Authority 2006).

Whereas it is undoubtedly important to encourage prevention of food contamination by OTA and other mycotoxins, these observations suggest that OTA is not likely to be an aetiological factor involved in BEN and associated UUC. OTA poisoning can, however, influence the metabolism of other carcinogens like AA in vivo (see Stiborová et al. 2015a).

### Organic chemicals from Pliocene lignite deposits

The so-called lignite hypothesis was formulated by scientists from the US Geological Survey in the 1990s (Feder et al. 1991; Orem et al. 1999). It is based on the geographical matching between the location of Pliocene lignite deposits in the Balkans and the location of endemic areas (Pavlovic 2013) and the assumption that toxic organic chemicals in lignite, or in weathered lignite, might be released by groundwater and hence contaminate drinking water wells. Although the concentrations of these organic compounds in well water are low, long exposure and/or accumulation in body tissues over time may result in adverse health effects, including symptoms of BEN (Bunnell et al. 2007; Pfohl-Leszkowicz et al. 2002; Tatu et al. 1998).

Drinking water used in endemic regions was thought to contain polycyclic aromatic hydrocarbons (PAHs), aromatic amines, phenols, and phthalates from the low-rank coals. Indeed, the presence of some of these compounds in groundwater samples from endemic villages has been described (Feder et al. 1991; Maharaj et al. 2014). Two water samples from endemic areas and one from a non-endemic area in Serbia showed high concentrations of naphthalene, fluorine, phenanthrene, and pyrene (Orem et al. 1999). Later it was shown that levels of aliphatic and aromatic compounds were higher in water samples in BEN areas than in those from non-endemic areas in Romania (Orem et al. 2002). Likewise, another study (Maharaj et al. 2014) indicated that compounds, such as benzenes, phenols, phthalates, polycyclic aromatic hydrocarbons, and/or lignin degradation compounds, occur in higher concentrations in extracts of endemic area Pliocene lignite sample. In contrast, a study by Voice et al. (2006) found no detectable levels of any of the 16 priority pollutants designated by the US Environmental Protection Agency were found in water samples throughout the Balkans, and no difference in waterborne concentrations of these pollutants between BEN and non-endemic villages (Voice et al. 2006). These findings suggest that the Pliocene lignite hypothesis is limited to the spatial association originally proposed in BEN (Maharaj 2014). Therefore, exposures of individuals living in endemic areas to the compounds from the lignite deposits are not relevant for the aetiology of BEN.

### Aristolochic acid

**Fig. 2:**
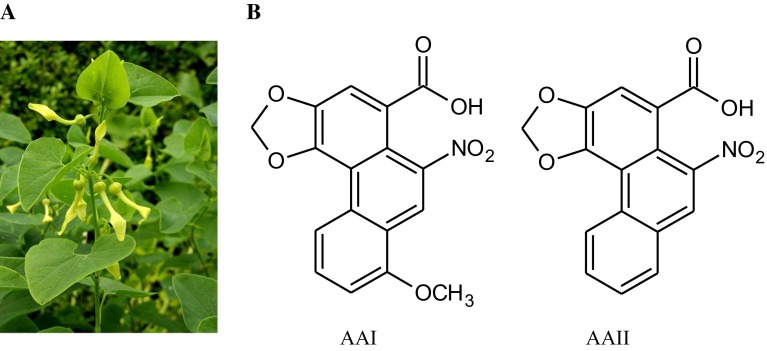
*Aristolochia clematitis* (**a**) and the formula of the major components of the AA plant product, aristolochic acid I (AAI) and aristolochic acid II (AAII) (**b**)

Over the last 10 years aristolochic acid (AA) has emerged as a causative factor of BEN, particularly the development of BEN-associated UCC. AA is the natural plant extract of both the *Aristolochia* and *Asarum* genera of the family Aristolochiaceae, in Europe especially *Aristolochia clematitis* (Heinrich et al. 2009). The extract consists of structurally related nitrophenanthrene carboxylic acids with AAI and AAII being the major components (Fig. 2). The AA hypothesis was proposed by Kazantzis and Ivic already in 1967 (Ivic 1969; Ivic and Lovriæ 1967; Kazantzis 1967) but was neglected for many decades.

It was proposed that contamination of the baking flour in endemic areas by seeds of the birthwort *Aristolochia clematitis* (Fig. 2) was the cause of BEN. The initial study of this theory was carried out by Ivic (1969). He found that these plants grew in local wheat fields as a weed and that its seeds comingled with wheat grain during the harvesting process. He administered *Aristolochia* seeds to rabbits that developed renal damage and speculated that human exposure to the toxic component of the *Aristolochia* seeds could occur through ingestion of bread prepared with flour derived from contaminated grain. Rabbits that were fed flour containing *Aristolochia clematitis* seeds developed nephropathy, which on a histological level resembled findings of BEN. Ivić even proved the carcinogenetic potential of the plant because rat used as experimental model developed sarcomas at the site of injection of aqueous extracts of *Aristolochia clematitis* (Ivic and Lovriæ 1967). Although these well-documented results provided evidence for the involvement of AA in BEN, Ivić’s observations were neglected for many decades till 1993.

**Fig. 3:**
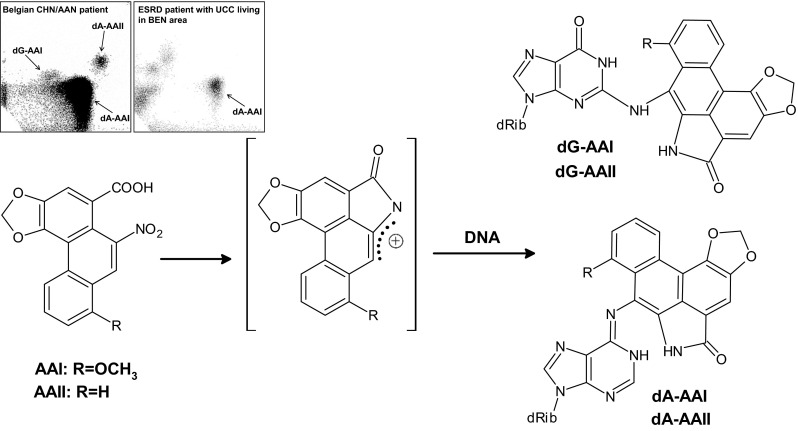
Metabolic activation and DNA adduct formation of aristolochic acid I (AAI) and II (AAII); 7-(deoxyadenosin-*N* ^6^-yl)aristolactam I or II (dA-AAI or dA-AAII), 7-(deoxyguanosin-*N* ^2^-yl)aristolactam I or II (dG-AAI or dG-AAII). *Inserts* Autoradiographic profiles of DNA adducts detected by ^32^P-postlabelling showing the analysis of renal tissue of an CHN/AAN patient in Belgium (Nortier et al. 2000) and a patient with end-stage renal disease (ESRD) and UCC living in an area endemic for BEN (Arlt et al. 2002b)

**Fig. 4:**
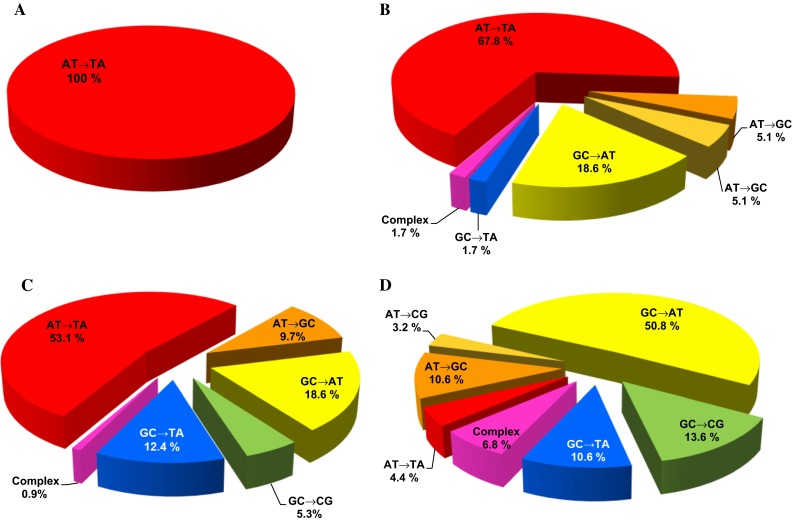
Mutation pattern in TP53 of UUC. Mutation data from human tumours were obtained from the IARC TP53 mutation database (http://www.p53.iarc.fr; R18 version). **a** TP53 mutation pattern in AAN-associated urothelial cancer in the United Kingdom (*n* = 1) (Lord et al. 2004). **b** TP53 mutation pattern in BEN-associated urothelial cancer in Croatia, Bosnia, Serbia (*n* = 59) (Grollman et al. 2007; Moriya et al. 2011). **c** TP53 mutation pattern in AAN-associated urothelial cancer in Taiwan (*n* = 113) (Chen et al. 2012). **d** TP53 mutation pattern in urothelial cancer not associated with AA exposure (*n* = 1127). Organs included: kidney, bladder, renal pelvis, ureter and other urinary organs. Morphology inclusion criteria: carcinoma not otherwise specified, carcinoma in situ not otherwise specified, dysplasia not otherwise specified, papillary carcinoma not otherwise specified, transitional cell carcinoma in situ, transitional carcinoma not otherwise specified and urothelial papilloma not otherwise specified

In that year, Vanherweghem et al. (1993) described the occurence of a novel renal disease that developed in hundreds of young Belgian women. The disease was initially described as Chinese herbs nephropathy (CHN), but later renamed aristolochic acid nephropathy (AAN) (Arlt et al. 2004; Debelle et al. 2008). At a single medical clinic in Brussels, ESRD developed in these women after receiving slimming pills including Chinese herbs. Cosyns first called attention to the unique renal histopathology of CHN exhibiting high similarity to BEN predominantly on morphological and clinical grounds (Cosyns et al. 1994; Cosyns 2003). It was proven that the slimming pills in Belgium contained Chinese herbs which were contaminated with nephrotoxic AA. Its presence in the slimming pills was the result of an accidental substitution of the prescribed herb *Stephania tetrandra* by *Aristolochia fangchi*, a plant species of the *Aristolochia* genus known to contain AA. Subsequently, UCC developed in nearly 50 % of CHN patients suffering from ESRD (Cosyns et al. 1998, 1999; Nortier et al. 2000), which again demonstrated high similarities of CHN with BEN. Importantly, specific AA-derived DNA adducts were found by the ^32^P-postlabelling method by Schmeiser and coworkers for the first time in renal and ureteric tissue of CHN patients (Arlt et al. 2004; Bieler et al. 1997; Lord et al. 2001, 2004; Nortier et al. 2000; Schmeiser et al. 1996) proving exposure to AA in these patients (Fig. 3). In 2004, one patient suffering from AAN showed a specific AAG to TAG transversion mutation (an A:T → T:A transversion) in codon 139 (Lys → Stop) of exon 5 in the tumour suppressor gene *TP53* (Fig. 4a) (Lord et al. 2004). These A:T → T:A transversion mutations were also found in a group of CHN patients with urothelial malignancy, but also other types of mutations were identified (Aydin et al. 2014). The apparent selectivity for mutations at adenine residues in AA-induced urothelial tumours is consistent with the high prevalence of the 7-(deoxyadenosin-*N* ^6^-yl)aristolactam I (dA-AAI) adduct in the target tissue of CHN patients. This adduct also shows a long persistance in renal tissue of these CHN patients and is still detectable decades after AA exposure (Schmeiser et al. 2014). The mutated adenine in codon 139 of *TP53* has the same neighbouring bases as in codon 61 (CAA) of the H-*ras* gene in experimental rodent models (rats, mice), where also characteristic A:T → T:A transversions have been found after AA treatment suggesting a sequence specific mechanism during mutation induction (Schmeiser et al. 1990, 1991; Wang et al. 2011, 2012). Because the observed A:T → T:A transversions in *TP53* are consistent with the known mutagenic specificity of AA (Schmeiser et al. 1990, 1991; Broschard et al. 1994), it was proposed that AA-induced A:T → T:A transversion mutations in *TP53* of urothelial tumours can be used as mechanistically relevant biomarkers of AA exposure in combination with specific AA-DNA adduct formation in urothelial tissue of these patients (Lord et al. 2004; Arlt et al. 2007). Mutations at these sites have not previously been associated with UUC and, thus, appear to be uniquely associated with exposure to AA (Arlt et al. 2007; Moriya et al. 2011; Olivier et al. 2012). These data indicated the molecular mechanism, whereby AA causes urothelial malignancy (Arlt et al. 2007; Gökmen et al. 2013).

These reports renewed scientific interest in the old hypothesis that AA is involved in the development of BEN. New investigations focused on the use of specific AA-DNA adducts as biomarkers of AA exposure using the ultra-sensitive ^32^P-postlabelling method. Both major AA components, AAI and AAII, are enzymatically reduced to reactive cyclic acylnitrenium ions that bind to the exocyclic amino groups of dA and dG to form AA-derived DNA adducts, 7-(deoxyadenosin-*N* ^6^-yl)aristolactam I or II (dA-AAI or dA-AAII) and 7-(deoxyguanosin-*N* ^2^-yl)aristolactam I or II (dG-AAI or dG-AAII) (Fig. 3) (Pfau et al. 1990a, b; Schmeiser et al. 1996, 1997; Stiborová et al. 1994, 2008a, 2011).

As described above, dA-AAI is the most abundant and persistent DNA adduct found in CHN patients (Arlt et al. 2001, 2002a; Bieler et al. 1997; Nortier et al. 2000; Schmeiser et al. 1996, 2014) and are therefore ideal robust biomarkers of AA exposure in BEN patients. The first molecular clue that BEN patients are exposed to AA was provided by Arlt and coworkers (Arlt et al. 2002b). They found that dA-AAI adducts were present in kidney tissue of some patients suffering from UCC and living in endemic areas of BEN (Fig. 3). The presence of AA-DNA adducts in renal tissue was later confirmed in larger cohorts and patients with definite diagnosis of BEN from endemic regions in Croatia, Serbia, and Bosnia; no AA-DNA adducts were found in patients with other forms of chronic renal disease or patients with UCC living in nonendemic areas of Croatia and Serbia (Grollman et al. 2007; Jelakovic et al. 2012). Similarly, Schmeiser et al. (2012) detected AA-DNA adducts (i.e. dA-AAI) in renal tissue of patients who underwent nephroureterectomy for UUC and resided for 17 years or longer in BEN villages in Romania.

Besides demonstrating exposure of BEN patients to AA by the detection of specific AA-DNA adducts, other approaches used the detection of A:T → T:A mutations in UCC of BEN patients as mechanistic biomarker of AA effect (Grollman et al. 2007; Jelakovic et al. 2012; Schmeiser et al. 2012). A high prevalence of A:T → T:A transversions in *TP53* was found in urothelial tumours of BEN patients originating from Croatia, Serbia, Bosnia, and Romania (Fig. 4b), a mutation type which is otherwise rare in urothelial tumours not associated with AA exposure (Fig. 4d). These findings provide a clear molecular link between AA exposure and the formation of BEN-associated UCC and also demonstrate that AA is the common aetiological agent for BEN and UCC across its numerous geographical foci.

Mutated adenines associated with A:T → T:A transversions after AA exposure are almost exclusively located on the non-transcribed strand of DNA (Sidorenko et al. 2012). Therefore, the authors postulated that this marked strand bias might be responsible for the selective low removal of dA-AAI adducts from the transcribed strand by transcription-coupled nucleotide excision repair (Moriya et al. 2011; Sidorenko et al. 2012). Resistance of dA-AAI adducts to global genomic repair reflects the inability of XPC-RAD23B to recognise and bind to these lesions in duplex DNA (Sidorenko et al. 2012). This failure of global genomic repair to excise AA-derived DNA adducts also may account for the persistence of these lesions in human tissues (Grollman 2013). Indeed this conclusion is in accordance with the detection of AA-DNA adducts (i.e. dA-AAI) in AAN patients even decades after exposure to AA (Schmeiser et al. 2014).

The slower progression towards ESRD and UUC development in BEN patients compared to CHN/AAN in Belgium is likely linked to lower doses of AA ingested by contaminated food produced in BEN villages as compared with the high dose of AA found in the herbal mixtures used by patients in the Belgian “slimming” clinic. The higher prevalence of women in the Belgian CHN/AAN cohort can be attributed to the fact that young women are more likely to attend such clinics (De Broe 2012).

All these studies provided crucial information on explaining the molecular mechanism of AAN/BEN-associated carcinogenesis (reviewed in Arlt et al. 2002a, 2007; Stiborová et al. 2008a; Schmeiser et al. 2009; Gökmen et al. 2013). As a result, the National Toxicology Program (2009) lists AA as carcinogenic to humans. The report states that “sufficient” scientific evidence is available to conclude that exposure to AA causes urothelial cancer in humans through formation of DNA adducts (specifically, through binding of the reactive metabolite with adenine) and the resulting transversion mutations in oncogenes and the tumour suppressor gene *TP53*. Likewise, in 2012 AA was classified as carcinogenic to humans (Group 1) by the IARC acting by a genotoxic mechanism (IARC 2012).

Unique features of the *TP53* mutation spectrum in AA-induced UUC, including the predominance of A:T → T:A transversions, were also found in Taiwanese patients with UUC (Fig. 4c) (Chen et al. 2012b, 2016). This study confirmed the hypothesis that the mutational signature of AA in *TP53* established in the context of UUC associated with BEN (Grollman et al. 2007; Moriya et al. 2011), is the same as that found in Taiwanese patients suffering from UUC (Chen et al. 2012b). Studies using human *TP53* knock-in (Hupki) mouse embryo fibroblasts (HUFs) (Kucab et al. 2010) to investigate mutations induced by AA in *TP53* experimentally not only showed that the HUF immortalisation assay captures the mutational signature of AA (i.e. mutation pattern) in *TP53* but also shares so-called hotspot *TP53* mutations (i.e. mutation spectrum) observed in BEN-associated UCC (Feldmeyer et al. 2006; Liu et al. 2004; Nedelko et al. 2009; Olivier et al. 2012). More recently, characteristic A:T → T:A transversion mutations were also observed in loci of other genes by whole-genome and exome sequencing analysing AA-associated UUC (Hoang et al. 2013; Poon et al. 2013). Whole-genome and exome sequencing was also applied to cells immortalised in vitro after AA exposure, leading to similar results (Nik-Zainal et al. 2015; Olivier et al. 2014).

The most important clinical lessons that can be learned from BEN and CHN/AAN is that these diseases are preventable with simple measures. With improved regulation of herbal medicines and prevention of exposure to AA this illness can be completely eradicated (Gökmen et al. 2013). Although herbal remedies containing AA have been banned in many countries worldwide, the risk of AA exposure due to botanicals remains high in many regions of the world (Gökmen et al. 2013). As a consequence AAN has become a global iatrogenic disease (Grollman 2013). From this point of view, an important advance in the ability to analyse AA-derived DNA adducts was recently achieved, because mass spectrometry has proved to be a highly sensitive, specific and robust analytical method (Schmeiser et al. 2014; Yun et al. 2012, 2013, 2015) that could provide an alternative to the ^32^P-postlabelling method (Schmeiser et al. 2013), which has been commonly used over the last decades to detect and quantify AA-DNA adducts in human biomonitoring. Mass spectrometry has the advantage that it provides direct structural information of the DNA adduct. The applicability of this approach has been demonstrated recently by analysing renal tissue from Romanian cancer patients. Renal cell carcinoma has not been reported in AAN patients previously but the Romanian patients unexpectedly showed high frequencies of A:T → T:A transversion mutations by whole-genome sequencing of the renal tumours, which is consistent with exposure to AA (Scelo et al. 2014). In a subsequent study using mass spectrometry, dA-AAI adducts were detected in the Romanian cases unambiguously demonstrating exposure to AA in these patients (Turesky et al. 2016). As these patients do not cover the Romanian population of the BEN area (Scelo et al. 2014), the source of AA exposure remains unclear in this cohort. However, using AA-DNA adducts as biomarker of exposure and the unique mutational signature of AA as biomarker of effect clearly identified AA as a aetiologic agent of these cancers.

## Confounding factors that may influence the development of BEN

Although exposure to AA is a causal factor for the development of BEN, other questions still need to be answered. Why do only 5–10 % of the residents in an endemic area develop BEN (Bamias and Boletis 2008; Tatu et al. 1998)? The same phenomenon has been observed in the Belgian AAN cohort; only 10–20 % of patients in the slimming clinic in Brussels developed AAN (Debelle et al. 2008). In the case of BEN, this cannot be attributed easily to preferential exposure of such a small group of the population to AA, but it could result also from other factors. This could include several endogenous factors such as the effectiveness of detoxification and/or bioactivation of AA, the expression levels of biotransformation enzymes involved in AA metabolism and their genetic and phenotypic polymorphisms, other genetic and/or epigenetic factors, and immunological changes. Indeed, although the theories on the aetiology of BEN mainly focused on environmental factors, recently particularly AA, confounding factors were also considered that may influence the molecular pathology of BEN.

## Genetic and epigenetic factors of BEN development

The familial pattern of BEN suggests a multifactorial nature of the aetiology of this disease which potentially includes genetic predisposition of individuals suffering from BEN (Toncheva and Dimitrov 1996). Indeed, combined effects of genetic and environmental factors might lead to the development of BEN, determining its clinical and epidemiological characteristics and disease progression. This hypothesis was investigated among various Bulgarian families, where family members suffered from BEN and included 4077 persons from 417 affected families (Toncheva et al. 1998). The authors concluded that all patients with BEN belonged to families. Interestingly, even residents from non-endemic villages which were identified to be members of BEN families that had migrated from the places they were born (i.e. villages in BEN areas) were diagnosed to suffer from BEN. Moreover, in this study epidemiological characteristics of the BEN disease indicated the involvement of genetic disorders, in which the proportion of the affected offspring was associated with the number of parents affected by BEN (Toncheva et al. 1998). Accordingly, the risk of developing BEN was much greater in first-degree relatives than second-degree relatives and was considerable weaker in distant relatives.

To resolve additional genetic factors that influence the development of BEN, cytogenetic investigations were carried out. These studies aimed to investigate the impact of chromosomal abnormalities on the occurrence of BEN and the frequent association with cancer (Stefanovic 1998; Toncheva et al. 1988, 1991; Tsoneva et al. 1985). It was shown that in healthy relatives of BEN patients born in non-endemic areas a specific BEN-associated locus exists in 3q25 (Stefanovic 1998; Toncheva et al. 1988; Toncheva and Dimitrov 1996). Alterations in 3q25 could also dictate genetic susceptibility for the development of BEN in relatives of patients having BEN (Toncheva and Dimitrov 1996). Other studies have suggested genes located in chromosome band 3q25–3q26 to be important for BEN; these genes encode for xenobiotic-metabolising enzymes, tumour suppressor proteins and proto-oncoproteins (Toncheva and Dimitrov 1996). Abnormality in 3q25 included the oncogenes *c*-*src* (cytoplasmic tyrosine kinase, CSK, 1q36), *raf*-*1* (murine leukaemia viral oncogene homolog 1, RAF1, 3p25) and *myb* (V-myb myeloblastosis viral oncogene homolog, MYB, 6q23) (Bamias and Boletis 2008; Toncheva et al. 1991; Toncheva and Dimitrov 1996). More recently, next generation sequencing (i.e. exome sequencing) demonstrated three mutant genes associated with the process of angiogenesis; *CELA1* (the gene of chymotrypsin like elastase-1), *HSPG2* (the gene of heparan sulphate proteoglycan 2), and *KCNK5* (the gene of potassium channel subfamily K member 5) (Toncheva et al. 2014). Therefore, the authors suggested that abnormal angiogenesis may be important in the molecular pathogenesis of BEN (Toncheva et al. 2014).

Epigenetic modifications may also influence the development of BEN. In a case–control study differentially methylated regions were identified which showed hypomethylation of the promoters of genes *HDAC11* (the gene of histone deacetylase 11)*, IL*-*17RA* (the gene of 17 receptor, alpha subunit)*, SECG61* (the gene of protein translocase complex, SecE/Sec61-gamma subunit) (Staneva et al. 2013). This suggests that dysregulation of genes involved in immunological responses could be a mechanism in BEN pathogenesis. Other epigenetic alterations included increased acetylation of histone lysine residues (i.e. H3 and H4 histones) in isolated urothelial cells of BEN patients (Kocic et al. 2014).

## Metabolism of aristolochic acid

Beside the route of uptake and dose of AA, metabolism dictates its biological effective concentration, thereby modulating disease development (i.e. BEN/AAN) and progression (i.e. urothelial malignancy). The metabolism of AA has been studied in several species including human.

**Fig. 5:**
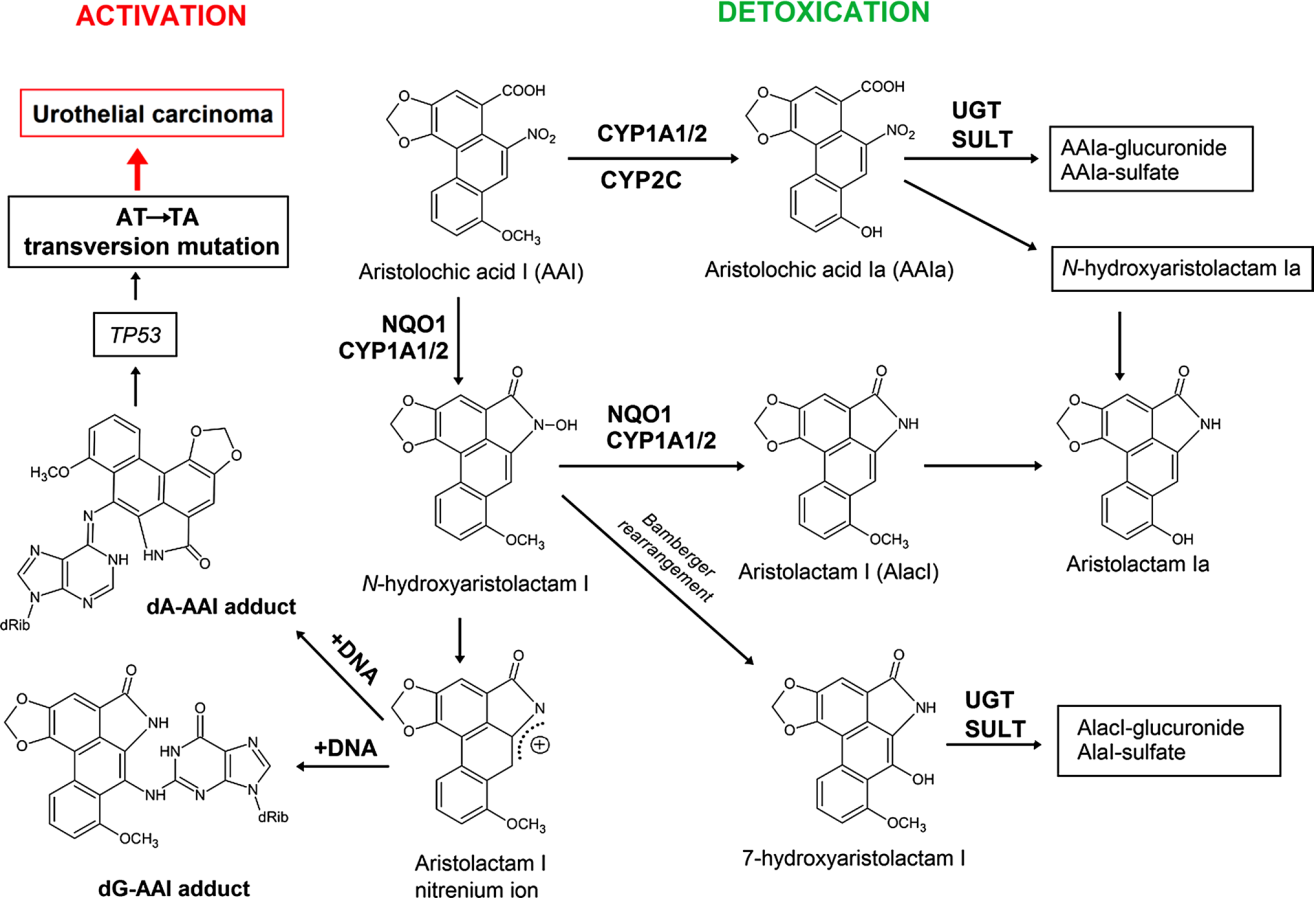
Activation and detoxication pathways of AAI. *dA*-*AAI* 7-(deoxyadenosin-*N* ^6^-yl)aristolactam I, *dG*-*AAI* 7-(deoxyguanosin-*N* ^2^-yl)aristolactam I, *CYP1A1/2* cytochrome P450 1A1 and 1A2, *CYP2C* cytochrome P450 2C, *NQO1* NAD(P)H:quinone oxidoreductase, *UGT* UDP glucuronosyltransferase, *SULT* sulfotransferase

AAI, the major component of the natural plant extract, is considered to be responsible for AA-mediated nephropathy. Although AAI might directly cause interstitial nephropathy (Shibutani et al. 2007), enzymatic activation of AAI to intermediates capable of binding to DNA is a necessary reaction leading to AA-mediated malignant transformation (Arlt et al. 2007; Grollman et al. 2007; Stiborová et al. 2008a, b, 2013b, 2014a, c). Both oxidative and reductive metabolites of AAI are formed in organisms after exposure to AAI and they are excreted in urine and faeces (reviewed in Arlt et al. 2002a; Stiborová et al. 2008a, b, 2013b). 8-Hydroxyaristolochic acid I (aristolochic acid Ia, AAIa) is the product of oxidative demethylation of AAI and considered a detoxification metabolite (Fig. 5) (Chan et al. 2006; Shibutani et al. 2010). Aristolactams I and II are the predominant products of AA metabolism in humans (Fig. 5) (Chan et al. 2006; Krumbiegel et al. 1987). AAI is reduced to *N*-hydroxyaristolactam I which is either further reduced to aristolactam I or rearranged to 7-hydroxyaristolactam I (Fig. 5) (Chan et al. 2007).

Interestingly, another AAI metabolite, mainly found in experimental animals, is aristolactam Ia. Two pathways can lead to the formation of aristolactam Ia: (i) through demethylation of aristolactam I, or (ii) through *O*-demethylation of AAI to AAIa that is then reduced to aristolactam Ia (Fig. 5). These suggested pathways are based on the finding that aristolactam I is formed in vitro under anaerobic conditions, while under aerobic conditions only AAIa is formed from AAI (Schmeiser et al. 1986). Hence, the in vivo oxygen concentration of tissues may influence the relative extent of nitroreduction and *O*-demethylation of AAI (Maier et al. 1987). A majority of AAI metabolites found in the urine and faeces in rodents are present as conjugates, as the *O*-glucuronide, the *O*-acetate and the *O*-sulphate esters of AAIa, and the *N*- and *O*-glucuronides of aristolactam Ia (with prevalence of the *N*-glucuronide) (Chan et al. 2006).

Initial reduction of AAI to *N*-hydroxyaristolactam I is the activation pathway responsible for the carcinogenic effect of AAI. Rearrangement of *N*-hydroxyaristolactam I to the corresponding 7-hydroxyaristolactam I or further reduction to aristolactam I should be considered as detoxication pathway, because both metabolites are excreted (Chan et al. 2006, 2007). Indeed, this conclusion was confirmed in rats where treatment with aristolactam I resulted in 50-fold lower levels of AAI-DNA adducts (i.e. dA-AAI and dG-AAI) in renal tissue than after AAI treatment (Dong et al. 2006). Aristolactam I has also be shown to be not toxic to mice (Sato et al. 2004). However, Li et al. (2010) have recently demonstrated that aristolactam I exhibits cytotoxicity in human proximal tubular epithelial HK-2 cells, causing S-phase arrest. No DNA adducts are generated from AAIa, 7-hydroxyaristolactam I, or aristolactam Ia in human and animal models, indicating that they are detoxification metabolites (reviewed in Stiborová et al. 2013b). No significant histological changes were found in renal tissue of mice treated with AAIa, again confirming that AAIa is a detoxification metabolite of AAI (Shibutani et al. 2010).

It is noteworthy that detoxification of AAI to AAIa is decreased by OTA. Combined administration of OTA and AA to rats increased AA genotoxicity (i.e. AA-DNA adduct formation) (Stiborová et al. 2015a), suggesting that OTA might, to some extent, enhance AA-induced urothelial malignancy in BEN. In contrast, heavy metals and phthalates, which are present at high concentrations in the drinking water of BEN Pliocene lignit areas (Maharaj et al. 2014), have no influence on AA metabolism (i.e. AAI detoxification) in vitro (Barta et al. 2015).

### Enzymes involved in metabolism of aristolochic acid

As mentioned above, one of the common enigmatic features of AAN and BEN is that only a few individuals exposed to AA suffer from nephropathy and cancer. The underlying mechanism(s) for this phenomenon still needs to be understood (Tatu et al. 1998; Bamias and Boletis 2008; Stiborová et al. 2013b). Besides differences in the accumulated dose of AA and the duration of AA intake (Nortier et al. 2000; Martinez et al. 2002), differences in the activities of enzymes catalysing the biotransformation of AA (detoxification and/or activation) could be the reason for an individual’s susceptibility (reviewed in Stiborová et al. 2008a, b, 2009, 2013b, 2014a, c). Many genes of enzymes metabolising AA are known to exist in variant forms or show polymorphisms resulting in different enzyme activities of the gene products. These genetic variations appear to be important determinants of cancer risk or other toxic effects of many xenobiotics (Stiborová et al. 2008a, b, 2009). The combination of polymorphic enzymes along with environmental exposure to AA may result in an increased risk for the development of BEN/AAN (Atanasova et al. 2005; Toncheva et al. 2004; Toncheva 2006; Chen et al. 2012a). Hence, research over the last two decades has aimed to identify the enzymes principally involved AA metabolism (reviewed in Stiborová et al. 2008a, b, 2009, 2013b, 2014a, c, 2015b).

The metabolic activation of AAI to an electrophilic cyclic *N*-acylnitrenium ion forming AAI-derived DNA adducts found in urothelial tissues of AA-exposed patients is mainly catalysed by cytosolic NAD(P)H:quinone oxidoreductase (NQO1) (Stiborová et al. 2002a, 2003, 2011, 2014a, c). The role of NQO1 in AAI nitroreduction was also proven in vivo using mouse and rat models (Chen et al. 2011; Stiborová et al. 2014a). Studies investigating the participation of enzymatically-catalysed conjugation reactions in AAI activation showed contrasting results. Using cytosolic fractions no participation of phase II conjugation enzymes in the bioactivation of AAI (i.e. AAI-DNA adduct formation) was found in cell-free systems in vitro (Martinek et al. 2011; Stiborová et al. 2011). These systems tested native enzymes present in human cytosols and human recombinant enzymes [i.e. sulfotransferases (SULT), mainly SULT1A enzymes, and *N*,*O*-acetyltransferases (NATs)]. These findings are consistent with another study where analysis using hepatic cytosols from several human donors showed that only NQO1 activity correlated with higher AAI-DNA adduct formation in vitro (Stiborová et al. 2003). On the contrary, Meinl et al. (2006) reported that expression of human SULT1A1 in bacterial and mammalian cells (Bendadani et al. 2014) enhanced the mutagenicity AA. Others showed that *O*-sulfonated and *O*-acetylated *N*-hydroxyaristolactam I and II readily form DNA adducts in vitro and that binding of *N*-hydroxyaristolactam I and II to DNA was stimulated by mouse cytosol in the presence of 3′-phosphoadenosine-5′-phosphosulfate, the cofactor for SULT enzymes (Sidorenko et al. 2014). Furthermore, human SULT1B1, SULT1A1, and SULT1A2 were capable of stimulating DNA adduct formation by *N*-hydroxyaristolactam I and II (Sidorenko et al. 2014). Hashimoto et al. (2016) indicated that bioactivation of AAI and *N*-hydroxyaristolactam I is dependent on SULT1A1 in human kidney (HK-2) and skin fibroblast (GM00637) cell lines in vitro. In contrast, studies in transgenic mice carrying the functional human *SULT1A1*–*SULT1A2* gene cluster and *Sult1a1(‒/‒)* mice showed that sulfo conjugation catalysed by human SULT1A1 and murine Sult1a1 does not play a role in the activation pathways of AAI and AAII in vivo (Arlt et al. 2016).

Human microsomal cytochrome P450 (CYP) enzymes are also capable of reducing AAI in vitro, with CYP1A2, and, to lesser extend, CYP1A1, being most efficient. Cytochrome P450 oxidoreductase (POR), another microsomal enzyme, plays only a minor role in AAI nitroreduction (Milichovský et al. 2016; Stiborová et al. 2001a, b, 2005a, b). Participation of these CYPs in the reductive activation of AAI was also demonstrated in rodents in vivo. Genetically modified mouse lines employed in these studies included hepatic reductase null (HRN) mice (Levová et al. 2011), *Cyp1a1(*−*/*−*)*, *Cyp1a2(*−*/*−*)* and *Cyp1a1/1a2(*−*/*−*)* mouse lines (Arlt et al. 2011a; Rosenquist et al. 2010). Using transgenic *CYP1A*-humanised mouse lines that carried functional human *CYP1A1* and *CYP1A2* genes and that lacked the mouse orthologous genes confirmed the importance of human CYP1A1 and CYP1A2 in AAI bioactivation in vivo (Stiborová et al. 2012). Since in human liver CYP1A1 is expressed at relatively low levels (Drahushuk et al. 1998; Stiborová et al. 2002b), its contribution to AAI activation in human liver is much lower than that of CYP1A2.

**Fig. 6:**
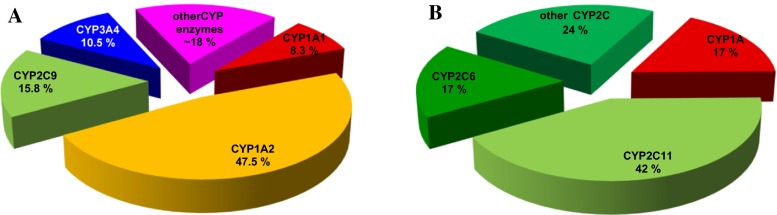
Contributions of CYP enzymes to AAIa formation in human (**a**) and rat livers (**b**)

It is important to note that CYPs of the 1A subfamily play a dual role in AAI metabolism. They are also the major enzymes oxidising AAI to AAIa under aerobic (i.e. oxidative) conditions in vitro (Levová et al. 2011; Rosenquist et al. 2010; Sistkova et al. 2008; Stiborová et al. 2011, 2012, 2015b). Other CYPs such as CYP2C (i.e. CYP2C8/9/19), CYP3A (i.e. CYP3A4/5), 2D6, 2E1 and 1B1 also form AAIa, but efficiency is more than one order of magnitude lower compared to CYP1A enzymes (Levová et al. 2011; Stiborová et al. 2012, 2015b). Because the liver is the most important organ responsible for CYP-catalysed xenobiotic biotransformation, the efficiencies of CYPs to oxidise AAI (i.e. detoxify AAI to AAIa) in human and rodent livers was studied in detail (Levová et al. 2011; Stiborová et al. 2012, 2015b). Human CYP1A2 followed by CYP2C9, CYP3A4, and CYP1A1 were the major enzymes contributing to AAI oxidation in human liver, while CYP2C and 1A were most important in rat liver (Fig. 6). Human CYP2E1, 2C8, and 2C19 partially contributed to AAI oxidation (≥1 %), whereas contributions of human CYP1B1, CYP2B6, CYP2D6, and CYP3A5 to AAI oxidation in human livers was negligible (Levová et al. 2011; Stiborová et al. 2012; 2015b).

The importance of mouse Cyp1a1/2 enzymes to catalyse the oxidative demethylation of AAI to AAIa was shown also in vivo, utilising the genetically modified mouse lines described above (Arlt et al. 2011a; Levová et al. 2011; Rosenquist et al. 2010; Xiao et al. 2008) and in mice where Cyp1a1/2 expression was enhanced by inducers (Xue et al. 2008). These studies showed that these mouse Cyps oxidise AAI to AAIa and protect these animals from AAI-induced acute renal injury (Rosenquist et al. 2010; Xiao et al. 2008). When the animals are not able to detoxify AAI through demethylation to AAIa, higher concentrations of unmetabolised AAI are available for bioactivation to form AAI-DNA adducts, or to cause renal injury. Indeed, high levels of AAI-DNA adducts were found in several organs of *Cyp1a*-knockout mouse lines, predominantly in the kidney (Arlt et al. 2011a; Levová et al. 2011; Rosenquist et al. 2010). In addition, an increase in AAI-mediated nephrotoxicity in mice lacking Cyp1a was observed in these animals (Rosenquist et al. 2010). Further, induction of CYP1A1/2 in rats led to an increase in AAI detoxification to AAIa, thereby reducing the actual amount of AAI available for reductive activation (Dračínská et al. 2016). This subsequently resulted in lower AAI-DNA adduct levels in the rat in vivo demonstrating that of the two opposing CYP1A1/2-catalysed reactions (i.e. oxidation and reduction of AAI), CYP1A1/2-mediated oxidative detoxification of AAI prevails in AAI metabolism in vivo, thereby suppressing levels of AAI-DNA adducts (i.e. AAI genotoxicity) (Dračínská et al. 2016). Even more importantly oxidation of AAI to AAIa by human CYP1A1 and 1A2 was also shown in vivo using *CYP1A*-humanised mouse lines (Stiborová et al. 2012).

The dual role of CYP1A1/2 in AAI metabolism can be explained by the fact that AAI can be a ligand substrate for human CYP1A1/2 enzymes at low oxygen concentrations, where AAI is reduced instead of being oxidised during the CYP-mediated reaction cycle (Jerabek et al. 2012; Milichovský et al. 2016; Stiborová et al. 2013b). Under aerobic (*i.e.* oxidative) conditions AAI is a classical substrate of CYP1A1/2 where during this process one atom of molecular oxygen is used to *O*-demethylate the methoxy group of AAI to generate AAIa (Stiborová et al. 2013b, 2015b). These experimental findings (Arlt et al. 2011a; Levová et al. 2011; Stiborová et al. 2005a, b, 2012, 2013b, 2015b) are supported by theoretical approaches (i.e. computational studies) (Jerabek et al. 2012; Milichovský et al. 2016; Stiborová et al. 2013b, 2014b, 2015b), indicating that, in addition to CYP1A1/2 expression levels, the in vivo oxygen concentration in tissues might affect the balance between AAI nitroreduction and demethylation, which in turn influences tissue-specific toxicity and carcinogenicity.

### Can genetic or phenotypic polymorphisms of enzymes metabolising AA contribute to the development of AAN and BEN? Still more questions than answers

The levels and activities of xenobiotic-metabolising enzymes catalysing the activation and detoxification of AA (e.g. NQO1, CYP1A1/2, CYP2C9, CYP3A4/5) depend on several factors such as their basal expression, regulation, induction, and/or inhibition (Rendic and DiCarlo 1997) as well as their polymorphisms (Atanasova et al. 2005). Expression and activities of enzymes involved in AA metabolism can differ in individuals due to a number of factors. All of them are inducible enzymes and expression can be modulated by environmental chemicals, drugs, and several hormones (reviewed in Rendic and DiCarlo 1997; Ross 2004; Ross et al. 2000). It is also noteworthy that exposure to AA itself can induce some of these enzymes (e.g. NQO1 and CYP1A1/2) (Arlt et al. 2011a; Bárta et al. 2014; Dračínská et al. 2016).

NQO1 expression is regulated by two distinct regulatory elements in the 5′-flanking region of the *NQO1* gene, the antioxidant response element (ARE) and the xenobiotic response element (XRE), involving ligand-activated aryl hydrocarbon receptor (AhR) (Jaiswal 2000; Ross 2004). ARE-mediated *NQO1* gene expression is increased by a variety of antioxidants, tumour promoters, and reactive oxygen species (ROS) (Li and Jaiswal 1994). ROS has been shown to be generated in several human cells in culture after AAI exposure (Yu et al. 2011; Zhu et al. 2012) suggesting that AAI-mediated ROS formation might be one mechanism by which AAI induces NQO1. However, this potential mechanism or other mechanisms that lead to NQO1 induction remain to be explored in future studies. It is noteworthy that the human XRE of *NQO1* shares significant homology with the XRE of human *CYP1A* (Nebert and Jones 1989; Nebert et al. 2000), another enzyme metabolising AA. *NQO1* and *CYP1A* are induced by many AhR ligands such as polycyclic aromatic hydrocarbons and azo dyes (Ross 2004; Stiborová et al. 2013a, 2013b; Yu et al. 2011). Moreover, as mentioned above, AAI is capable of inducing NQO1 protein levels and enzyme activity in rodent models, thereby enhancing its own genotoxicity (Arlt et al. 2011b; Dračínská et al. 2016; Levova et al. 2012; Stiborová et al. 2001c, 2012, 2015a). Thus it can be anticipated that NQO1 expression is also induced in individuals exposed to AA.

Induction of CYP3A4/5 is regulated by the constitutively activated receptor (CAR) and the pregnane X receptor (PXR), drugs, environmental substances and glucocorticoids have been shown to induce CYP3A4/5 (Rendic and DiCarlo 1997). It has been proposed that enhanced expression of CYP3A5 caused by exposure to such inducers may phenocopy the effects of the high expression allele CYP3A5*1 (Burk et al. 2004). However, the effect of AAI on CYP3A4/5 expression has not been investigated as yet.

Genetic polymorphisms in *NQO1*, *CYP1A1/2*, *CYP2C9*, and *CYP3A4/5* may also impact on an individual’s susceptibility to AA. The role of some genetic polymorphisms of biotransformation enzymes [*NQO1*, *CYP1A1*, *CYP2D6*, *CYP3A4/5*, *NAT1/2*, *glutathione*-*S*-*transferase (GST) GSTT1*, *GSTM1*, *GSTP1* and *GSTA1*] has already been examined in BEN/AAN patients (Atanasova et al. 2005; Chen et al. 2012a; He et al. 2005; Reljic et al. 2014; Stefanovic et al. 2006; Toncheva et al. 1998, 2004; Toncheva 2006; Wang et al. 2009). Among the enzymes metabolising AAI, polymorphisms in the human *NQO1* gene were reported to be important in BEN patients (Toncheva et al. 2004; Toncheva 2006). The genotype NQO1*2 (C609T) predisposed BEN patients to the development of UCC (Toncheva 2006). This finding appears to be opposite to what one might expect, given the importance of NQO1 in AAI activation; however, diminished NQO1 metabolism of AAI could lead to an enhanced body burden which might lead to an increased risk of tumourigenesis over time (Levova et al. 2012). Among a group of AAN patients, no significant associations between the polymorphisms of the *NQO1 C609T* gene and disease risk were observed (Chen et al. 2012a), suggesting that the *NQO1* variants evaluated in their study do not play a decisive role in the development of AAN.

Higher risk for BEN was observed in individuals carrying the CYP3A5*1 allele G6989 (Atanasova et al. 2005; Toncheva 2006). The CYP3A5 enzyme, which is expressed also in human kidney (Rendic and DiCarlo 1997), has been shown to be capable of both activating (i.e. DNA adduct formation) (Levová et al. 2011) and detoxifing (i.e. formation of AAIa) of AAI in vitro (Stiborová et al. 2012, 2015b). However CYP3A5 is much less effective in these reactions than CYP1A. No relationships between *CYP1A1* polymorphisms and AAN have been found (Chen et al. 2012a) and alterations in the *CYP1A2* gene have not been investigated. Likewise, CYP3A4*1B and *CYP2D6* genotypes do not modify the risk of developing BEN (Atanasova et al. 2005). To date phenotyping of CYP enzymes was studied only with debrisoquine as the marker substrate of CYP2D6 and showed that the distribution among patients with BEN/UUT was associated with a predominance of extensive debrisoquine hydroxylation and a lack of poor metabolisers (Nikolov et al. 1991). However, given the minor role of CYP2D6 in AA metabolism the interpretation of these findings need be taken with caution.

Among the polymorphisms of further biotransfomation enzymes tested previously, the distribution frequency of GSTT1 null genotype among AAN patients was significantly higher than in controls and associated with a 1.7-fold increased risk of developing AAN (Chen et al. 2012a). It has also been shown that the GSTM1 wild-type allele is associated with BEN; significantly lower prevalence of the GSTM1 deletion homozygotes among BEN patients suggested that individuals bearing the GSTM1-null genotype were better protected (Andonova et al. 2004). However, to evaluate the biological significance of these findings, it is necessary to know whether the GST conjugation enzymes participate in the metabolism of AA. Recently, Reljic et al. (2014) also analysed the association between common *GSTA1*, *GSTM1*, *GSTT1*, and *GSTP1* polymorphisms and susceptibility to BEN. They found that *GSTA1* was significantly associated with a higher risk of BEN. Interestingly, using in silico simulation the authors suggested that GSTA1-1 might be involved in catalysing the formation of glutathione conjugates of OTA metabolites (i.e. ochratoxin hydroquinone) (Reljic et al. 2014).

## Conclusions and recommendations

The data summarised in this review emphasises that chronic intoxication with AA, a plant product of *Aristolochia* species, is the main causal agent for the development of BEN and particularly BEN-associated UUC. This conclusion is based on its similarities to the pathology of AAN, the detection of specific AA-derived DNA adducts in renal tissue of BEN patient and the dominance of the A:T → T:A transversion mutations in *TP53* in BEN-associated UUC (mutational signature) which are otherwise rare in individuals with UCC not exposed to AA.

Nevertheless, there is still at least one enigmatic feature of BEN that need to be resolved. As not all individuals exposed to AA suffer from this disease, besides differences in the cumulated dose of AA and the duration of AA intake, differences in the activities of the enzymes catalysing the biotransformation of AA may predispose certain residents in BEN areas impacting on an individual’s susceptibility. However, the real impact of these enzymes on AA-induced nephropathy and UUC in humans still remains to be understood. Studies evaluating associations of genetic polymorphisms of the enzymes metabolising AA and the risk of developing AAN, BEN, and UUC have brought controversial results. Further, investigations focusing only on genetic polymorphisms without taking the expression levels of the enzymatically active proteins into account may offer only limited conclusions. We believe that the analyses of the expression levels of enzymes metabolising AA and their phenotyping in AAN and BEN patients will bring greater advances in determining their real contribution to the development of AA-induced nephropathies and cancer risk among these patients.

Because the distribution of *Aristolochia* species is worldwide and the use of medicinal herbal remedies containing AA is still widespread, AA might be the cause of yet unrecognised nephropathies and UUC.

## Abbreviations

AA: Aristolochic acids
AAI: Aristolochic acid I
AAIa: Aristolochic acid Ia (8-hydroxyaristolochic acid I)
AAII: Aristolochic acid II
AAN: Aristolochic acid nephropathy
AhR: Aryl hydrocarbon receptor
ARE: Antioxidant response element
BEN: Balkan endemic nephropathy
CHN: Chinese herbs nephropathy
CKD: Chronic kidney disease
CYP: Cytochrome P450
dA-AAI: 7-(Deoxyadenosin-*N* ^6^-yl)aristolactam I
dA-AAII: 7-(Deoxyadenosin-*N* ^6^-yl)aristolactam II
dG-AAI: 7-(Deoxyguanosin-*N* ^2^-yl)aristolactam I
dG-AAII: 7-(Deoxyguanosin-*N* ^2^-yl)aristolactam II
dG-OTA: OTA-deoxyguanosine adduct
EFSA: European Food Safety Authority
ESRD: End-stage renal disease
HRN: NADPH:cytochrome P450 reductase null
HSPG2: Heparan sulphate proteoglycan 2
HUFs: Hupki mouse embryo fibroblasts
IARC: International Agency for Research on Cancer
NAT: *N,O*-Acetyltransferase
NQO1: NAD(P)H:quinone oxidoreductase
OTA: Ochratoxin A
ROS: Reactive oxygen species
SULT: Sulfotransferase
UGT: UDP glucuronosyltransferase
UUC: Upper urothelial cancer
XRE: Xenobiotic response element
